# Integrating Multi-Source and Multi-Temporal UAV Observations to Improve Wheat Yield Prediction Using Machine Learning

**DOI:** 10.3390/plants15091345

**Published:** 2026-04-28

**Authors:** Chen Chen, Jiajun Liu, Yao Deng, Rui Guo, Weicheng Yao, Tianle Yang, Weijun Zhang, Tao Liu, Xiuliang Jin, Wei Xiong, Dongsheng Li

**Affiliations:** 1 Jiangsu Hilly Region Zhenjiang Agricultural Science Research Institute, Jurong 212400, China; 2 College of Agriculture, Yangzhou University, Yangzhou 225009, Chinatliu@yzu.edu.cn (T.L.); 3 Institute of Crop Sciences, Chinese Academy of Agricultural Sciences, Beijing 100081, China; 4 Development Center of Science and Technology, Ministry of Agriculture and Rural Affairs, Beijing 100176, China

**Keywords:** UAV remote sensing, wheat yield estimation, multi-temporal data fusion, machine learning, plant phenomics

## Abstract

Accurate yield estimation is vital for precision wheat management and breeding. Traditional methods based on single growth stages or single-source data cannot capture cumulative growth effects, limiting prediction accuracy. UAV remote sensing provides high-resolution, multi-source, and multi-temporal data, enabling improved non-destructive yield estimation. In this study, UAV-based multispectral and RGB imagery were collected at six key growth stages, and vegetation indices, texture, and color features were extracted to develop yield prediction models using RF, XGBoost, and KNN under single- and multi-temporal scenarios. The results showed that red-edge-based vegetation indices were highly sensitive to wheat yield and outperformed texture- and color-based features. Multi-feature fusion further improved prediction accuracy at key growth stages, particularly during booting and flowering (R^2^ = 0.53–0.67). Compared with single-temporal models, multi-temporal data fusion significantly enhanced yield estimation accuracy, achieving a maximum R^2^ of 0.72 by integrating data from the late-jointing, booting and flowering stages. Among the algorithms, XGBoost and KNN exhibited superior accuracy and stability across most growth stages. Overall, these results demonstrate that integrating UAV-based multi-source and multi-temporal remote sensing data effectively improves the accuracy and robustness of wheat yield estimation, providing valuable technical support for precision agriculture and phenotyping-assisted breeding.

## 1. Introduction

Wheat is one of the most important staple crops sustaining global food supply and food security. Accurate, efficient, and timely prediction of wheat yield is of great significance for cultivar breeding, optimization of agronomic management, and the safeguarding of food security [1]. Achieving this goal relies on systematic investigation and continuous improvement of wheat yield estimation approaches.

Methods for wheat yield assessment can generally be classified into direct measurement and indirect estimation. Traditional direct measurement methods are typically based on harvesting, threshing, and weighing, which are labor-intensive and time-consuming, making them unsuitable for large-scale or high-throughput applications. In contrast, indirect estimation approaches mainly rely on remote sensing–based prediction models, particularly machine learning models that utilize remotely sensed phenotypic traits such as plant height, biomass, and canopy structure [2,3]. For example, MODIS satellite imagery with a spatial resolution of 500 m has been used to construct vegetation indices combined with machine learning algorithms, enabling national-scale wheat yield estimation (R^2^ = 0.72–0.76) in the United States [4]. Similar studies have further demonstrated the feasibility of remote sensing–based modeling approaches for large-scale yield assessment and macro-level agricultural management [5,6]. In recent years, research attention has gradually shifted from large-scale monitoring to plot-scale field phenotyping, aiming to improve yield estimation accuracy by exploiting higher spatial resolution proximal sensing data. Proximal sensing refers to the acquisition of target information using sensors deployed on near-ground platforms. Compared with contact-based measurements and satellite remote sensing, proximal sensing provides data with higher spatial, temporal, and spectral resolutions. Among proximal sensing platforms, unmanned aerial vehicles (UAVs) offer high flexibility and mobility, as flight altitude and acquisition timing can be adjusted according to specific monitoring requirements. Consequently, UAV-based remote sensing has shown outstanding performance in improving operational efficiency and monitoring accuracy. To a certain extent, UAV remote sensing bridges the gap between ground-based and satellite observations in terms of efficiency and spatial resolution, providing strong support for crop growth monitoring and yield estimation [7]. UAV-based remote sensing has been successfully applied to yield estimation of crops such as rice, wheat, and rapeseed [8,9,10].

In yield prediction studies, multi-source data integration can provide richer and more complementary information. Sensors including hyperspectral, multispectral, and red–green–blue (RGB) cameras have been widely used for crop yield estimation. Vegetation indices (VIs) derived from multispectral imagery have been successfully applied to wheat yield prediction. For example, the best single-variable model, based on the in-season estimated yield, achieved an R^2^ of 0.62 with an RMSE of 0.72 t ha^−1^ [11]. Meanwhile, color features and classical color indices extracted from RGB imagery can effectively characterize variations in canopy leaf color, nutritional status, and growth conditions. Previous studies have shown that the digital numbers of RGB channels exhibit strong correlations with wheat yield, with correlation coefficients exceeding 0.75 [12]. In addition, texture features, which comprehensively reflect canopy structure, leaf distribution, and the spatial heterogeneity of leaf color [13], are capable of capturing structural variations in crop canopies at the spatial scale. During crop growth, differences in nitrogen uptake and utilization influence leaf color and canopy architecture, thereby altering texture patterns in remote sensing imagery, which in turn are associated with yield variability. Therefore, integrating multiple types of remote sensing features is expected to enhance the accuracy and robustness of crop yield prediction.

However, in the context of multi-source data integration, traditional linear models or empirical index-based approaches often fail to fully exploit high-dimensional feature information. Although physically based models have also been applied to crop yield prediction (e.g., RMSE = 0.51 t ha^−1^), they typically require complex input parameters and are mainly suited for large-scale applications, limiting their adaptability to small experimental plots with high management heterogeneity [14]. In contrast, machine learning models can effectively integrate multiple yield-related features without explicitly describing complex physiological processes, making them powerful tools for crop yield estimation and feature contribution analysis [15]. Previous studies have compared various machine learning approaches, including random forest (RF), artificial neural networks (ANN), support vector machines (SVM), and stepwise regression (SWR). For instance, ANN models outperformed both SVR and RF in cotton yield prediction, achieving an R^2^ of 0.72 on the test dataset as early as 70 days after planting. In wheat yield prediction, random forest models have demonstrated superior performance, with R^2^ values ranging from 0.53 to 0.79 across different growth stages [16,17]. However, these findings also indicate that model performance is highly dependent on the dataset and application scenario. Nevertheless, the use of machine learning approaches that integrate multi-temporal spectral indices, texture features, and color features for wheat yield estimation remains insufficiently explored and warrants further in-depth investigation.

Moreover, final crop yield is closely associated with nutrient accumulation and canopy structural dynamics across multiple growth stages. For example, Zhu et al. [8] utilized UAV imagery acquired at the jointing, heading, and grain-filling stages of wheat to develop yield prediction models based on vegetation indices. Their results indicated that the heading to grain-filling stage is the optimal period for yield estimation using single-stage VIs, with multi-temporal models achieving R^2^ values up to 0.70. Similarly, Gong et al. [10] used UAV-based multispectral imagery collected at the early flowering stage of rapeseed and reported R^2^ values ranging from 0.33 to 0.81 using several commonly used vegetation indices. Maimaitijiang et al. [18] integrated RGB, multispectral, and thermal infrared imagery acquired at the early pod-setting stage to estimate soybean yield, achieving an R^2^ of 0.72. These studies demonstrate that high spatial resolution optical imagery acquired at one or more key phenological stages can effectively improve crop yield prediction accuracy [9,19]. Therefore, a systematic evaluation of multi-temporal remote sensing feature fusion strategies is necessary to fully explore their potential for early prediction of wheat yield. Based on the above considerations, this study aims to: (1) extract and analyze multiple types of spectral and image-derived features throughout the wheat growth period and identify yield-sensitive features; (2) develop wheat yield prediction models by integrating vegetation indices, color indices and texture features using machine learning approaches; and (3) evaluate the effects of multi-temporal remote sensing feature fusion on the performance of wheat yield prediction models.

## 2. Results

### 2.1. Spatiotemporal Variability of Multi-Source Remote Sensing Features Across Key Growth Stages

**Figure 1:**
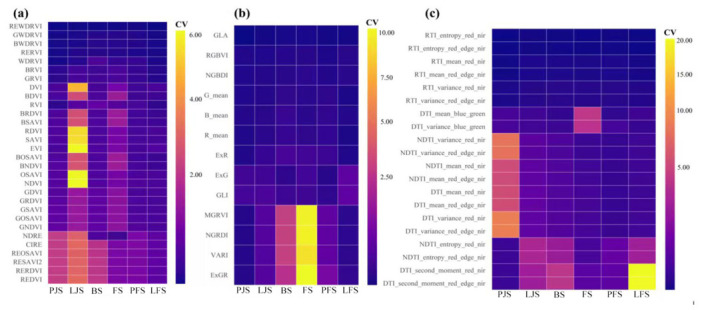
Coefficient of variation (CV) of spectral (**a**), color (**b**), and textural (**c**) features across observation dates. Notes: PJS, LJS, BS, FS, PFS and LFS represent the Pre-jointing, Late-jointing, Booting, Flowering, Pre-filling, and Late-filling stages, respectively.

To quantify field-level heterogeneity, the mean (mean), standard deviation (sd), and coefficient of variation (CV) of each feature were calculated across 28 plots for each observation date. Three CV-based heatmaps were constructed to illustrate the temporal evolution of within-date variability for vegetation indices, color indices, and texture indices, respectively (Figure 1). Red-edge indices (e.g., CIRE, NDRE, REDVI, and REOSAVI) consistently showed relatively higher CV values (often exceeding 1.5). In these cases, the inflated CV primarily reflects the amplification effect caused by small denominators rather than unusually large absolute dispersion. A similar phenomenon was observed on Late-jointing, when several indices (e.g., EVI, NDVI, OSAVI) exhibited near-zero, resulting in extremely large CV values (e.g., CV > 7 for EVI). These results highlight the strong sensitivity of CV to mean magnitude and underscore the need to interpret CV jointly with mean and sd, particularly during early or transitional phenological stages.

RGB channel mean features (R_mean, G_mean, and B_mean) displayed relatively stable behavior across dates, indicating limited plot-to-plot variability in brightness-related attributes (Figure 1b). In contrast, several ratio- or difference-based color indices (e.g., ExGR, MGRVI, NGRDI, and VARI) showed pronounced increases in CV on specific dates. As a result, although color indices can capture subtle canopy color differences, their relative stability varies substantially across time, warranting careful screening prior to downstream modeling.

GLCM-derived texture indices (TIs) generally showed low to moderate variability across plots (Figure 1c), RTI-based entropy metrics were highly stable throughout the season, indicating strong spatial consistency of entropy-related texture information. RTI_mean features also remained relatively stable (CV = 0.04–0.08 across dates). In contrast, DTI or NDTI metrics involving mean, variance, or second-moment statistics exhibited substantially larger dispersion on certain dates. For example, on Pre-jointing, DTI_mean__RE_NIR (and DTI_mean_R_NIR) had CV = 3.87, while DTI_variance_RE_NIR (and DTI_variance_R–NIR) reached CV = 6.89; similarly, NDTI_variance_RE_NIR showed CV = 6.07. The most pronounced case occurred on Late-filling, where DTI_second_moment_RE–NIR (and DTI_second_moment_R_NIR) exhibited CV = 23.17.

### 2.2. Correlation Analysis Between Multi-Source Remote Sensing Feature and Wheat Yield Across Key Growth Stages

**Figure 2:**
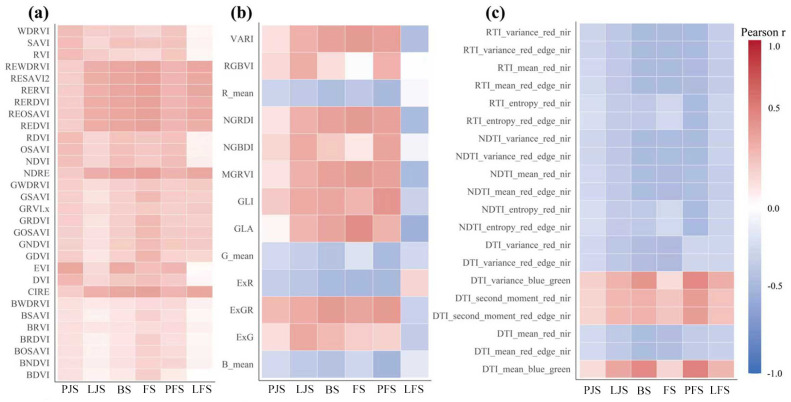
Pearson correlations between yield and spectral (**a**), color (**b**), and textural (**c**) features across observation dates. Notes: PJS, LJS, BS, FS, PFS and LFS represent the Pre−jointing, Late−jointing, Booting, Flowering, Pre−filling, and Late−filling stages, respectively.

Across the six observation dates, yield–feature correlations exhibited clear temporal dynamics and differed by feature type (Figure 2). Vegetation indices generally showed stronger and more consistent positive associations with yield than color and texture indices. In particular, red-edge–based indices peaked in mid-season, with NDRE reaching the highest correlation on Flowering (r = 0.69) and remaining strong on Booting (r = 0.64), indicating that canopy chlorophyll sensitivity was most informative around this period. Color indices displayed moderate correlations that strengthened toward late April, highlighted by GLA on Flowering (r = 0.54), whereas several color metrics changed sign by Late-filling (predominantly negative), suggesting phenology-dependent spectral–yield relationships.

Texture-related predictors exhibited relatively weak correlations with yield at early growth stages; however, their explanatory power increased markedly as the season progressed. From late April onward, both conventional GLCM texture features and the derived texture indices (TIs) showed substantially strengthened associations with yield, with multiple metrics reaching absolute correlation coefficients of approximately |r| ≈ 0.45–0.55 around Pre-filling and Late-filling. Notably, texture indices constructed from variance, homogeneity, entropy, and second-moment components—particularly those involving red, red-edge, and near-infrared band combinations—consistently ranked among the top correlated variables at later stages. These results indicate that integrative texture indices (NDTI, DTI, and RTI) effectively amplify yield-relevant spatial information embedded in single-band texture features. Overall, the observed temporal pattern suggests that canopy structural heterogeneity, as captured by texture-based descriptors, becomes increasingly relevant to yield formation during the mid-to-late growth stages. Based on these results, the top ten features most strongly correlated with yield were selected from each category—vegetation indices, color indices, and texture indices—for subsequent model development.

### 2.3. Linear Analysis of Yield to Optimal Remote Sensing Feature at Key Observation Dates

Univariate linear regression was applied to quantify the individual relationships between grain yield and the candidate color, spectral, and texture features selected in Section 2.2. For each observation date and feature category, the variable with the highest R^2^ was retained, and its regression parameters are summarized in Table 1. The explanatory power of single features exhibited a clear temporal dependence. At early observation dates (Pre- and Late- jointing), all feature categories showed weak relationships with yield (R^2^ generally < 0.20). From early April onward, spectral indices—particularly the red-edge–based NDRE—demonstrated substantially stronger associations, reaching an R^2^ of 0.41 and 0.47 in the Booting and Flowering stages, respectively. In contrast, color and texture features showed moderate and more variable relationships, with their highest R^2^ values (approximately 0.25–0.34) occurring at later growth stages. Overall, the univariate analysis indicates that yield sensitivity to individual features increases with crop development and is strongly stage-dependent, with vegetation indices providing the most informative signals during mid-season.

### 2.4. Machine Learning-Based Yield Estimation Using Multi-Source Features at Key Observation Dates

To assess the predictive capability of single-date observations, regression models were independently developed for each acquisition date using four feature strategies: spectral, color, texture, and their combination. For each date-strategy pair, five machine learning algorithms were evaluated, and the best-performing model was selected based on the highest coefficient of determination (R^2^) (Table 2). Overall, single-date regression performance was moderate and exhibited strong temporal dependence. At the early growth stage (Pre-jointing), all feature strategies showed limited explanatory power (R^2^ < 0.20), indicating insufficient yield-related information in early-season single-date observations. As crop development progressed, prediction accuracy improved substantially. The strongest single-date performance was observed at the booting stage, a critical mid-season growth period. On this date, spectral features alone yielded an R^2^ of 0.49, whereas the combined feature strategy further improved model performance, achieving an R^2^ of 0.67 and the lowest normalized RMSE (0.14). In addition, the model demonstrated high computational efficiency, with a runtime of 0.28 s and memory usage of 74.80 MB. These results highlight the complementary value of integrating spectral, color, and texture features.

On Flowering, the combined strategy remained the best-performing approach (R^2^ = 0.53), followed by spectral and color features, suggesting sustained sensitivity of canopy spectral responses during this period. In contrast, during the later stages (Pre-filling and Late-filling), the predictive capability of spectral features declined markedly (R^2^ = 0.11–0.12). During these stages, texture-based features consistently outperformed spectral features, although color features achieved the highest single-date accuracy, with R^2^ values of 0.27 and 0.30 on Pre-filling and Late-filling, respectively.

Across all observation dates, the combined feature strategy generally provided robust performance and achieved the highest or near-highest accuracy during the key mid-season stages. These findings suggest that, although single-date observations are inherently limited and sensitive to acquisition timing, the integration of complementary spectral, color, and texture features can effectively enhance the robustness of yield estimation across different growth stages. While models based on multi-type feature integration required slightly longer computational time, the runtime remained around 0.3 s, with memory usage not exceeding 75 MB, indicating a favorable balance between predictive performance and computational efficiency.

### 2.5. Integrated Yield Estimation Model Based on Multi-Source and Multi-Temporal UAV Observations

Compared with the single-date modeling results, incorporating multi-date observations substantially improved yield estimation accuracy, demonstrating the critical role of temporal information in capturing crop growth dynamics. Across the 20 three-date combinations evaluated, all feature strategies benefited from temporal aggregation; however, the magnitude of improvement varied markedly among spectral, color, texture, and combined feature sets. As summarized in Table 3, the highest overall performance was achieved using spectral features combined with the XGBoost model. The optimal configuration consisted of the three-date combination of Late-jointing, Booting, and Flowering, achieving an R^2^ of 0.72 and an nRMSE of 0.13. The model also exhibited acceptable computational efficiency, with a runtime of 0.56 s and memory usage of 88.60 MB. Two additional spectral-based combinations—Late-jointing, Booting, and Pre-jointing and Booting and Flowering and Pre-filling—also achieved strong performance (R^2^ = 0.70 and 0.66, respectively), with nRMSE values consistently below 0.15. Notably, these models required slightly lower computational resources, with runtimes below 0.55 s and memory usage under 88 MB. These top-performing combinations were all centered on the Late-jointing to early-filling period, corresponding to rapid vegetative growth and canopy expansion, indicating that spectral features acquired during this window provide highly complementary and yield-relevant information when integrated across time.

Color-based strategies exhibited comparable but generally lower predictive capability than spectral-based models. The top-performing color-based combinations reached a maximum R^2^ of 0.33 (nRMSE = 0.22, Computation time = 0.49 s, Memory usage = 85.80 Mb) using XGBoost, with optimal date combinations also concentrated around late March to late May. While color indices benefited from multi-date integration, their overall explanatory power remained substantially lower than that of spectral features. Texture-based strategies showed moderate but clearly improved performance under the multi-date framework compared with single-date results. The best texture-based configurations, all relying on XGBoost or SVM-RBF models, achieved R^2^ values between 0.39 and 0.43, with nRMSE values around 0.22–0.24. Notably, the inclusion of early-season (8 March) observations together with mid-season dates enhanced the predictive contribution of texture features, suggesting that temporal changes in canopy structural heterogeneity can partially compensate for their limited sensitivity at individual dates.

The combined feature strategy consistently produced intermediate performance, with the best three-date configuration (Late-jointing, Booting, and Flowering) achieving an R^2^ of 0.54 and an nRMSE of 0.17. The model required relatively higher computational resources, with a runtime of 0.96 s and memory usage of 132.40 MB. Although multi-source integration improved robustness relative to single-date models, the combined strategy did not surpass the best spectral-only configurations, suggesting that spectral features dominate the yield-related signal under multi-temporal conditions.

### 2.6. Feature Importance Analysis of Optimal Yield Estimation Models

**Figure 3:**
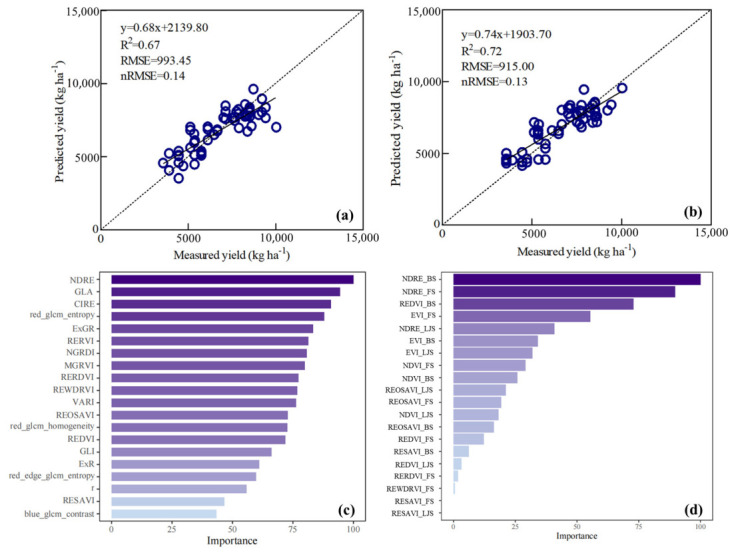
Validation results of the optimal wheat yield prediction models based on single−date (**a**) and multi−date (**b**) spectral information. Relative importance of input features under the single−date (Booting stage; (**c**)) and multi−date (**d**) model. Notes: LJS, BS, and FS represent the late-jointing stage, booting stage, and flowering stage, respectively.

The results indicate that, under the single-date modeling framework, the optimal model was achieved on Booting stage using XGBoost with a combined feature strategy integrating spectral, texture, and color indices (Figure 3a). In addition, for the multi-date modeling framework, the best performance was obtained by the XGBoost model that fused spectral indices from three key periods of (Late-jointing, Booting, and Flowering) (Figure 3b). To further explore the relative contributions of different features, feature importance analysis was conducted for both single-date and multi-date modeling frameworks.

Under the single-date modeling strategy, feature importance exhibited a mixed dominance of spectral indices, color indices, and texture features (Figure 3c). Among all predictors, the red-edge-based vegetation index such as NDRE showed the highest relative importance. Several color indices, including GLA, ExGR, NGRDI, and MGRVI, also ranked highly, highlighting the relevance of visible-band information for characterizing canopy color and physiological condition at a single time point. In addition to spectral and color features, texture metrics derived from GLCM analysis contributed substantially under the single-date framework. Texture features such as red_glcm_entropy, red_glcm_homogeneity, red_edge_glcm_entropy, and blue_glcm_contrast appeared among the top-ranked predictors, suggesting that spatial heterogeneity and canopy structural patterns provide complementary information when temporal context is limited. Overall, the single-date feature importance pattern reflects a balanced reliance on spectral response, canopy color, and spatial texture information.

The multi-date modeling framework revealed a markedly different feature importance pattern (Figure 3d). The results showed that the top five most important features were NDRE_BS, NDRE_FS, REDVI_BS, EVI_FS, and NDRE_LJS. The NDRE index exhibited exceptionally high importance across two different periods, indicating its stable predictive capability for wheat yield. Overall, features from Booting demonstrated higher importance than those from jointing, suggesting that spectral information from the later growth stages (e.g., Booting to Flowering) has greater explanatory power for yield formation. Additionally, indices such as EVI and NDVI also contributed to varying degrees, reflecting that the fusion of multiple indices can comprehensively capture crop growth conditions, thereby enhancing the model’s robustness and prediction accuracy.

## 3. Discussion

### 3.1. Advantages and Limitations of UAV Remote Sensing in Crop Information Collection

UAV platforms exhibit clear advantages over ground-based sensing systems and manual field surveys in terms of data acquisition efficiency. Previous studies have shown that large-scale spectral data collection using ground-based platforms may require several tens of hours [20], whereas UAVs can rapidly cover tens of hectares of farmland within a single flight. This capability makes UAVs particularly suitable for rapid yield assessment and the analysis of spatial heterogeneity in medium- to large-scale agricultural fields. In addition, UAV-based high-resolution imagery often enables higher prediction accuracy. In this study, the proposed approach achieved a maximum R^2^ of 0.72, demonstrating the strong potential of UAV data for yield estimation. Satellite remote sensing also provides a convenient means for large-scale, non-destructive, and near-real-time data acquisition. For example, Gong et al. [21] proposed the Temporal-Aware Spatial Interaction Transformer (TASI-Transformer) for crop yield prediction using multisensor satellite imagery, achieving an R^2^ of 0.51. Similarly, Jhajharia [22] reported R^2^ values ranging from 0.57 to 0.67 using satellite imagery combined with machine learning methods for wheat yield prediction, confirming the feasibility of satellite-based approaches. However, satellite data generally suffer from relatively low spatial resolution. This limitation is particularly pronounced in regions such as southern China, where agricultural fields are often small, fragmented, and irregularly distributed in hilly terrains, thereby constraining the applicability of satellite-based yield estimation. To address this issue, recent studies have explored the integration of UAV and satellite data, using high-resolution UAV imagery as an intermediate bridge to train models based on lower-resolution satellite data. Compared with satellite-only approaches (R^2^ = 0.67), such integration strategies have achieved improved prediction accuracy (R^2^ = 0.76) [23]. This approach effectively leverages the high spatial resolution of UAV data while mitigating the mismatch between satellite observations and ground-measured yield caused by coarse resolution.

Despite the promising potential of UAV-based multi-source remote sensing for crop yield estimation, several challenges remain for large-scale application. First, at the hardware level, the acquisition, maintenance, and operation costs of high-performance UAV platforms and multi-sensor systems are relatively high, limiting their accessibility, particularly for smallholder farming systems. Second, multi-temporal UAV data acquisition and processing are highly dependent on GNSS positioning accuracy. Although the integration of RTK technology can significantly improve spatial accuracy and image registration, it also increases system complexity and operational costs. Furthermore, the limited battery capacity and power systems of multi-rotor UAVs constrain flight endurance, creating efficiency bottlenecks in large-area applications. UAV operations are also subject to regional airspace regulations, which further restrict their flexible deployment and scalability [24]. From the perspective of data processing and application, although the machine learning models developed in this study exhibited high computational efficiency (with runtimes below 0.60 s and memory usage of approximately 90 MB for the optimal model), the continuous growth in data volume and sample size will impose increasing demands on data storage, computation, and management. This poses challenges for hardware infrastructure and technical capacity. In this context, cloud computing platforms and automated data processing pipelines are considered effective solutions for reducing technical barriers and improving data utilization efficiency [25]. More importantly, UAV platforms fundamentally serve as data acquisition tools. Their practical value in crop yield estimation ultimately depends on the ability to transform multi-source remote sensing data into robust models with clear physiological interpretability. Therefore, the development of crop yield inversion methods and intelligent modeling strategies tailored to UAV multi-source data remains a critical scientific challenge that requires further investigation.

### 3.2. Wheat Yield Estimation Based on Multi-Source Remote Sensing Features Using Machine Learning

The construction of high-accuracy wheat yield estimation models relies on a thorough understanding of the quantitative relationships between multi-source remote sensing features—such as vegetation indices, texture features, and color indices—and the yield formation process. The performance differences among vegetation indices across growth stages can be attributed to their sensitivity to crop physiological and structural characteristics. In particular, red-edge-based vegetation indices showed superior performance during the late-jointing to flowering stages. This is mainly because the red-edge region is highly sensitive to chlorophyll content, which is closely related to nitrogen status and photosynthetic capacity during periods of rapid vegetative growth [26]. During these stages, wheat exhibits high leaf nitrogen concentration and active biomass accumulation, resulting in strong absorption in the red region and a pronounced shift in the red-edge position. Consequently, red-edge indices are more effective in capturing subtle variations in canopy physiological status, leading to improved yield prediction performance [27]. Regarding texture features, the DTI index showed relatively strong correlations with wheat yield, particularly for texture feature combinations constructed from near-infrared, red-edge, and visible (green and blue) bands. These results suggest that texture information can capture canopy structural characteristics and spatial heterogeneity in crop distribution from a spatial perspective, which is consistent with previous findings reported for biomass and yield estimation in crops such as tea plants, wheat, and rice [1,28,29]. In terms of color features, color indices (CIs) such as ExGR and VARI exhibited relatively strong correlations with wheat yield. RGB-based color indices enhance and combine visible-band information, enabling effective characterization of physiological status and nutrient conditions reflected by leaf color variation, particularly in relation to nitrogen availability [30,31]. However, because RGB information is more sensitive to illumination conditions and background interference, its standalone application for yield estimation generally results in limited accuracy.

From a phenological perspective (Figure 2), spectral features acquired between Late-jointing and Pre-filling showed stronger correlations with final yield, indicating that canopy spectral characteristics during the period from booting to grain filling possess greater explanatory power for wheat yield formation. This period coincides with the critical stages of grain number and thousand-kernel weight determination, during which canopy structure and physiological status exert decisive influences on final yield. The yield estimation results based on different feature types (Table 1) indicate that models constructed using vegetation indices generally achieved the best performance (R^2^ = 0.41–0.47), followed by texture feature–based models (R^2^ = 0.21–0.34), while color feature–based models exhibited relatively lower accuracy (R^2^ = 0.22–0.29). Building on these findings, this study further explored multi-source feature fusion strategies by integrating vegetation indices, texture features, and color indices. The fused models achieved moderate performance improvements at certain key stages (e.g., Booting and Flowering), with R^2^ values ranging from 0.53 to 0.67; however, the overall improvement was limited. Compared with the results reported by Zhang et al. [28], who achieved higher accuracies (R^2^ = 0.75–0.81) in estimating wheat leaf area index and dry matter accumulation using random forest–based multi-feature fusion, the yield prediction accuracy obtained in this study was relatively lower. This discrepancy can be primarily attributed to differences in research objectives and prediction targets. While Zhang et al. [28] focused on instantaneous growth indicators during the growing season, the present study aims to perform forward-looking prediction of final yield based on remote sensing observations acquired at key phenological stages. During the period between remote sensing acquisition and final yield formation, multiple sources of uncertainty- such as soil nutrient availability, meteorological variability, and differences in management practices-can cumulatively influence yield, thereby increasing modeling complexity and limiting prediction accuracy.

In terms of model development, this study employed five machine learning algorithms, including Random Forest (RF), XGBoost, and K-Nearest Neighbors (KNN), to estimate wheat yield. It should be acknowledged that the number of ground-measured yield samples used for model training was relatively limited (n = 28). However, the primary focus of this study lies in the integration of multi-source features and multi-temporal information rather than reliance on large sample sizes. Specifically, a high-dimensional and information-rich feature space was constructed, the top ten vegetation indices, texture features, and color indices selected based on their correlation with yield, as well as multi-temporal data integration across six key wheat growth stages (pre-jointing, late-jointing, booting, flowering, pre-filling, and late filling). Such a feature configuration enhances the representation of crop growth dynamics, thus improving the model’s ability to capture spatial variability in yield. The model evaluation results indicate that, across most growth stages, XGBoost and KNN consistently outperformed the other algorithms in terms of R^2^ and RMSE (Table 2). The superior performance of XGBoost can be attributed to its gradient boosting framework, which iteratively fits residuals to capture complex nonlinear relationships. This mechanism enables effective extraction of intrinsic associations between multi-source remote sensing features and yield, while its regularization strategies help mitigate overfitting, an important consideration when dealing with high-dimensional and potentially collinear features. In contrast, KNN operates based on local similarity in the feature space, making predictions by referencing neighboring samples. This non-parametric approach is particularly advantageous in scenarios with limited sample sizes and relatively smooth feature distributions, as it does not rely on strong assumptions about the underlying data distribution. Its stable performance in this study suggests that local feature continuity plays a meaningful role in explaining yield variability. From a methodological perspective, the complementary strengths of XGBoost and KNN are noteworthy. While XGBoost excels at capturing global nonlinear patterns, KNN provides a robust mechanism for modeling local relationships. This complementarity implies that machine learning approaches capable of simultaneously leveraging global and local information may offer enhanced performance in multi-source, multi-temporal remote sensing-based yield estimation. Similar observations have been reported in previous studies, further supporting the effectiveness of such hybrid or complementary modeling strategies [1,32].

### 3.3. Improving Wheat Yield Estimation Through Multi-Temporal Remote Sensing Data Fusion

Crop yield formation is a typical cumulative and time-dependent process jointly driven by the evolution of crop physiological status across different growth stages and by dynamic environmental conditions. Remote sensing imagery acquired at a single time point can only represent the instantaneous growth status of crops at a specific phenological stage and therefore cannot fully capture the cumulative effects of multi-stage information throughout the yield formation process [1]. The results of this study demonstrate that multi-temporal remote sensing data fusion offers clear advantages for wheat yield estimation. By integrating multi-temporal remote sensing information acquired on Late-jointing, Booting, and Flowering, the R^2^ of the yield prediction model increased to 0.72, which was substantially higher than that achieved using single-date models. This improvement indicates that multi-temporal data can effectively integrate spectral and structural characteristics of wheat at different critical growth stages, thereby enhancing the stability and accuracy of yield estimation. During the wheat growth cycle, the contributions of different phenological stages to final yield vary considerably. The period from late March to late April corresponds to the late-jointing stage through the booting–grain filling stages, during which changes in canopy structure, leaf area index, and photosynthetic capacity directly affect grain number formation and dry matter accumulation, making this a critical phase for determining final yield [33]. Multi-temporal remote sensing data fusion enables the simultaneous capture of canopy phenotypic characteristics across these key growth stages, thereby avoiding the loss of important information or biases that may arise from suboptimal selection of a single observation date. Fu et al. [19] reported that wheat yield estimation based on random forest models integrating NDVI from the jointing, heading, flowering, and grain filling stages achieved significantly higher accuracy (R^2^ = 0.78, RMSE = 1030 kg ha^−1^) than models based on a single growth stage. Their findings are highly consistent with those of the present study, further validating the effectiveness of multi-temporal remote sensing information for wheat yield estimation. From a feature-level perspective, multi-temporal data fusion helps strengthen yield-related signals while reducing the influence of random noise on model performance.

As shown in Figure 3, the model exhibits a certain degree of systematic bias in yield prediction, characterized by slight overestimation for low-yield samples and underestimation for high-yield samples. This tendency may be attributed to the reduced sensitivity of UAV-derived spectral, texture, and color features under conditions of extremely low or high biomass, which in turn weakens the model’s ability to discriminate extreme yield values. Such regression-to-the-mean behavior is commonly observed in data-driven models when the feature space provides limited separability at the distribution tails. To address this limitation, future research should focus on increasing sample diversity and expanding data coverage under extreme conditions, thereby improving the representation of yield variability across the full spectrum. In addition, the integration of more diverse data sources-such as structural, meteorological, or soil-related information-may further enhance model sensitivity to extreme cases. Incorporating multi-year, multi-ecological region, and multi-variety datasets will also be essential for improving model robustness and generalizability. Furthermore, the results of this study demonstrate that the integration of multi-temporal data from the late-jointing, booting, and flowering stages alone is sufficient to achieve reliable early-stage yield prediction. This finding suggests that relatively high prediction accuracy can be attained during the early to mid-growth stages, substantially enhancing the practical applicability of the approach. Compared with predictions made at later growth stages, early- and mid-season yield estimation provides more actionable and forward-looking information for agricultural management, enabling optimized input allocation, improved harvest planning, and more informed market decision-making.

Although multi-source feature integration improved model robustness compared to single-date modeling, its predictive performance did not surpass that of the best spectral-only models (Table 3). This result indicates that, under multi-temporal conditions, spectral features remain the dominant source of information for characterizing tea yield, while texture and color features provide only limited incremental benefits. Previous studies have demonstrated that spectral features exhibit stronger robustness and temporal stability across different growth stages and sensing conditions, as they are directly linked to plant biochemical and physiological properties [34]. Spectral responses at different growth stages exhibit varying sensitivities to environmental stress, nutrient availability, and canopy structural changes. Consequently, multi-temporal features form complementary relationships within the model, enhancing its ability to identify key factors governing yield formation [35]. Particularly in years characterized by high climatic variability, multi-temporal remote sensing information can partially offset the adverse effects of extreme weather events on single-date imagery, thereby improving the robustness and applicability of yield estimation models. At the modeling level, multi-temporal remote sensing data fusion shows strong compatibility with machine learning approaches. Nonlinear machine learning models, such as random forest and gradient boosting algorithms, are capable of effectively extracting complex interactions among multi-temporal features and fully exploiting the combined contributions of different growth stages to final yield. The results of this study indicate that models incorporating multi-temporal features consistently outperformed single-date models in terms of R^2^ and RMSE, demonstrating that the inclusion of temporal information significantly enhances model generalization capability. These findings further confirm the advantages of combining multi-temporal remote sensing data with machine learning techniques for crop yield estimation.

Despite the significant potential of multi-temporal remote sensing data fusion for improving wheat yield estimation, several challenges remain for practical implementation. First, the acquisition of multi-temporal data relies on stable imaging conditions and high observation frequency, making it susceptible to constraints imposed by cloud cover, precipitation, and limited operational windows, which can compromise data continuity and completeness [24]. Second, multi-temporal feature integration substantially increases data dimensionality, placing higher demands on model training and feature selection. Without effective feature selection or dimensionality reduction strategies, redundant information may be introduced, thereby increasing the risk of overfitting. Moreover, a unified theoretical framework for quantifying the relative contributions of remote sensing features from different growth stages to yield formation is still lacking. This limitation hinders a deeper mechanistic understanding of multi-temporal data fusion and restricts the interpretability of modeling results. Future research should therefore focus on developing optimal fusion strategies for multi-temporal data, such as phenology-driven feature weighting approaches that explicitly account for the varying importance of different growth stages. In addition, the synergistic integration of multi-source remote sensing data—including optical, radar, and thermal infrared observations—offers considerable potential for improving model robustness and sensitivity [31]. The incorporation of auxiliary information, such as meteorological variables, soil properties, and field management practices, is also expected to enhance both model interpretability and predictive accuracy, thereby providing more reliable support for regional-scale yield forecasting and precision agriculture decision-making. From a modeling perspective, time-series approaches such as Long Short-Term Memory (LSTM) networks and Temporal Convolutional Networks (TCN) have demonstrated strong capability in capturing temporal dependencies in sequential data. However, their performance typically depends on high-frequency and continuous observations, which may not always be feasible in UAV-based data acquisition scenarios. To address this limitation, future studies should aim to construct comprehensive datasets encompassing multiple years, regions, crop varieties, and temporal sampling frequencies, thereby improving model robustness and generalizability. It is also important to note that multi-temporal UAV data acquisition significantly increases operational costs compared to single-date observations. In this context, the results of this study indicate that a single-date model based on the integration of spectral, texture, and color features can still achieve a relatively high accuracy (R^2^ = 0.67). Although this performance is slightly lower than that of multi-temporal models, it represents a favorable trade-off between accuracy and cost, particularly for real-time agricultural applications such as fertilization management. Finally, while the models developed in this study are computationally efficient, with low memory usage and fast runtime, challenges related to model scalability and deployment are likely to emerge as data volume and model complexity increase. Future efforts should therefore focus on model optimization through feature reduction and model compression techniques to improve efficiency under resource-constrained conditions. The integration of edge computing devices and cloud-based platforms could further enable rapid data processing and real-time prediction, thereby enhancing the deployability and practical applicability of UAV-based yield estimation systems in operational agricultural environments.

## 4. Materials and Methods

### 4.1. Study Area

**Figure 4:**
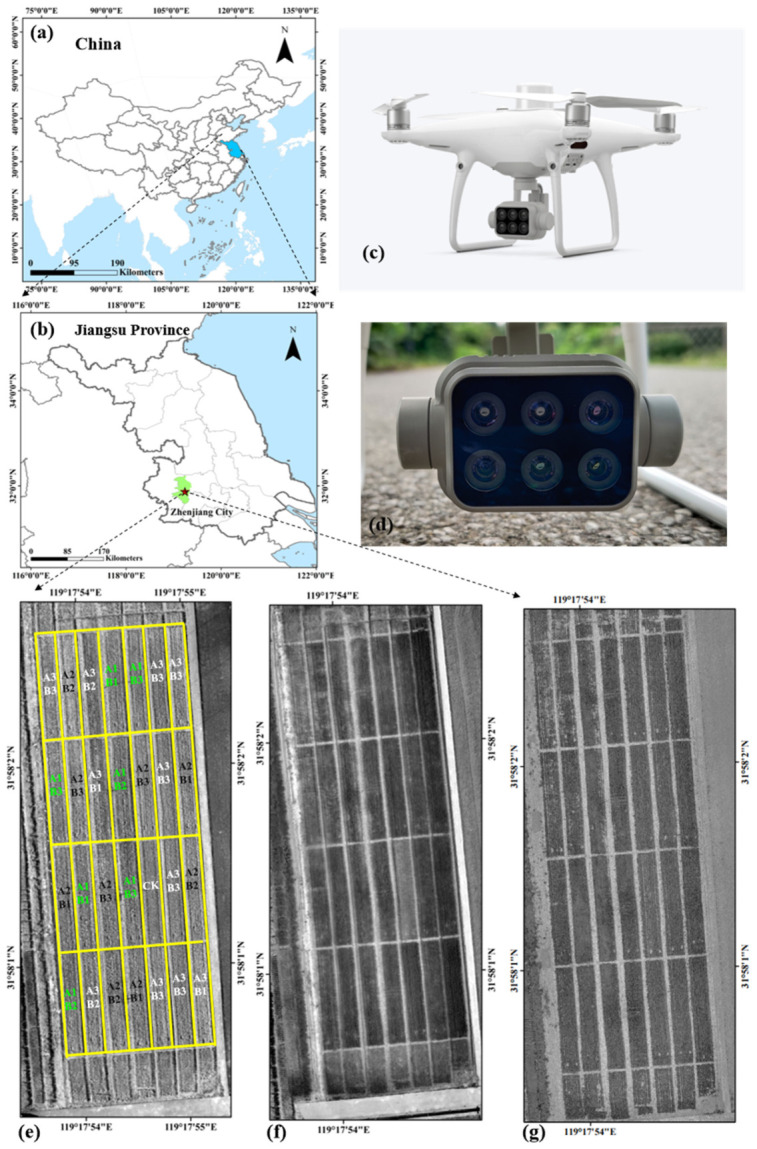
Overview of the study site (**a**,**b**); the UAV platform and spectral camera (**c**,**d**); and representative images of wheat plots showing NIR spectral reflectance (**e**), red features (**f**), and secondmoment texture features (**g**).

The field experiment was conducted during the 2022–2023 growing seasons in Jiangsu Province, eastern China (Figure 4). The winter wheat cultivar Zhenmai 12 was used. To establish a wide range of wheat yield levels, four nitrogen (N) application treatments were designed, including a zero-N control and three N fertilization rates of 180 (A1), 225 (A2), and 270 (A3) kg N ha^−1^. Under each N rate, three different basal-to-topdressing fertilization ratio schemes were applied, corresponding to basal, tillering, jointing, and booting stages, with ratios of 3:1:3:3 (B1), 5:1:2:2 (B2), and 7:1:2:0 (B3), respectively. Each treatment was replicated three times, as illustrated in Figure 1. The plot size was 16 m^2^, and all experiments were arranged in a randomized complete block design with three replications. Granular urea (46% N) was used as the nitrogen fertilizer. Phosphorus fertilizer was applied before sowing at a rate of 105 kg P_2_O_5_ ha^−1^ in the form of Ca(H_2_PO_4_)_2_, while potassium fertilizer was applied at 135 kg K_2_O ha^−1^, split equally between pre-sowing and the jointing stage. All plots were planted at a local standard density of 2.70 million seedlings ha^−1^ and managed following standard agronomic practices to avoid water, weed, and insect stresses. Irrigation was applied once during the sowing period to ensure uniform germination when rainfall was insufficient.

### 4.2. UAV-Based Spectral Data Acquisition and Feature Extraction

UAV imagery was acquired at six key wheat growth stages: pre-jointing (8 March), late-jointing (28 March), booting (6 April), flowering (28 April), pre–grain filling (6 May), and late grain filling (27 May). The intervals between successive observations were approximately 10–20 days, capturing the dynamic changes across critical phenological stages during wheat growth. Unmanned aerial vehicle (UAV) imagery was acquired using a DJI Phantom 4 remote sensing platform (DJI Innovation Technology Co., Ltd., Shenzhen, China) to collect high-resolution canopy information of wheat plants. The UAV was equipped with a multispectral camera comprising five spectral bands: blue (B; 450 ± 16 nm), green (G; 560 ± 16 nm), red (R; 650 ± 16 nm), red-edge (RE; 730 ± 16 nm), and near-infrared (NIR; 840 ± 26 nm). Each multispectral sensor had a field of view (FOV) of 62.7°, with an effective spatial resolution of 2 megapixels (1600 × 1300 pixels). In addition, the UAV carried an RGB camera with a 1/2.9-inch CMOS sensor, providing a resolution of 12 megapixels (4000 × 3000 pixels) and an FOV of 84°.

UAV flights were conducted between 11:00 and 14:00 local time under clear-sky conditions with low or negligible wind to minimize illumination variability and motion-induced artifacts. The flight altitude was set to 60 m above ground level, resulting in a ground sampling distance of approximately 4 cm. Forward and side overlap rates were fixed at 75% and 70%, respectively, to ensure sufficient image redundancy for orthomosaic generation.

Both multispectral and RGB images were processed using Pix4Dmapper 4.8 Ag software (Pix4D SA, Prilly, Switzerland) to generate georeferenced orthomosaics of the experimental area. Radiometric calibration was performed using standard reflectance panels with nominal reflectivities of 5%, 10%, 20%, and 40%. The orthomosaics were further georeferenced using evenly distributed ground control points (GCPs), whose coordinates were measured with a CHCNAV K90 GNSS receiver (Shanghai Huace Navigation Technology Co., Ltd., Shanghai, China).

Spectral reflectance values for each band were extracted at the sampling locations from the calibrated multispectral orthomosaics using ENVI 5.2 software (Exelis Visual Information Solutions, Boulder, CO, USA). Based on these reflectance values, a total of 28 vegetation indices (VIs) commonly used for crop growth monitoring and vegetation analysis were calculated (Table 4).

For RGB imagery, mean digital number (DN) values of the red (Rg), green (Gg), and blue (Bg) channels were extracted for each sampling plot using ENVI 5.2. These DN values (ranging from 0 to 255) were normalized to obtain standardized color components r, g, and b as follows:

(1) $$r=R_{g}/\left(R_{g}+G_{g}+B_{g}\right)$$

(2) $$g=G_{g}/\left(R_{g}+G_{g}+B_{g}\right)$$

(3) $$b=B_{g}/\left(R_{g}+G_{g}+B_{g}\right)$$

where Rg, Gg, and Bg represent the mean digital number values of each plot derived from RGB images, which range from 0 to 255.

### 4.3. Spectral Feature Extraction and Preprocessing

#### 4.3.1. Vegetation Index (VI) Extraction from Multispectral Imagery

Vegetation indices (VIs) were derived from calibrated multispectral reflectance data to characterize canopy greenness, biomass, and physiological status. Reflectance values of the near-infrared (NIR), blue (B), green (G), red (R), and red-edge (RE) bands were extracted for each plot from the UAV multispectral orthomosaics.

Based on these band reflectance values, a comprehensive set of vegetation indices was calculated, including ratio-based, difference-based, normalized difference, soil-adjusted, and wide dynamic range indices. To ensure numerical stability and avoid undefined values, all indices involving division operations were computed using a safe division strategy, whereby index values were set to missing when the denominator approached zero.

Specifically, vegetation indices were grouped according to the spectral band combinations involved: (i) NIR–Blue indices, including BRVI, BNDVI, BDVI, BRDVI, BSAVI, BOSAVI, and BWDRVI; (ii) NIR–Green indices, including GRVI, GNDVI, GDVI, GRDVI, GSAVI, GOSAVI, and GWDRVI; (iii) NIR–Red indices, including RVI, NDVI, DVI, RDVI, SAVI, OSAVI, and WDRVI; (iv) NIR–Red edge indices, including RERVI, NDRE, REDVI, RERDVI, RESAVI, REOSAVI, and REWDRVI. The mathematical definitions and references for all vegetation indices are provided in Table 4. All VI values were calculated at the plot level and subsequently used as candidate features for machine learning modeling.

#### 4.3.2. Color Index (CI) Extraction from RGB Imagery

Color indices (CIs) were derived from high-resolution UAV RGB imagery to capture canopy color information related to leaf pigment composition and vegetation condition. For each sampling plot, mean digital number (DN) values of the red (Rg), green (Gg), and blue (Bg) channels were extracted from the RGB orthomosaic images. To reduce the influence of illumination variability and brightness differences, the DN values were first normalized to obtain relative color components r, g, and b using the total RGB intensity. Based on these normalized components, a series of widely used color indices was computed, including excess color indices, normalized difference indices, and ratio-based indices. Specifically, the calculated color indices included Excess Green (ExG), Visible Atmospherically Resistant Index (VARI), Normalized Green–Red Difference Index (NGRDI), Green Leaf Index (GLI), Normalized Green–Blue Difference Index (NGBDI), Excess Red (ExR), Excess Green minus Excess Red (ExGR), Green–Red Vegetation Index (GRVI), Green Leaf Algorithm (GLA), Modified Green–Red Vegetation Index (MGRVI), and RGB Vegetation Index (RGBVI). These indices have been demonstrated to be effective in capturing subtle variations in canopy color and vegetation status. The complete list of color indices and their mathematical formulations is summarized in Table 4. All CI values were calculated at the plot level and integrated into the feature dataset for subsequent analysis.

#### 4.3.3. Texture Feature Extraction

Texture features were extracted from the UAV multispectral imagery to characterize the spatial heterogeneity and structural patterns of the tea canopy. The gray-level co-occurrence matrix (GLCM) approach was adopted, as it captures second-order statistical relationships between neighboring pixels and has been widely used in vegetation texture analysis. Prior to texture computation, each multispectral band (blue, green, red, red-edge, and near-infrared) was processed independently. GLCM texture features were calculated using ENVI 5.2 software with a moving window size of 3 × 3 pixels to capture fine-scale canopy variations while minimizing excessive spatial smoothing. To balance computational efficiency and texture discrimination capability, the gray-level quantization was set to 32 levels.

For each band, GLCMs were computed in four directions (0°, 45°, 90°, and 135°), corresponding to pixel offsets of (0,1), (1,1), (1,0), and (1,−1), respectively. The directional GLCMs were averaged to reduce orientation dependence. Eight commonly used GLCM texture metrics were derived, including mean (MEA), variance (VAR), homogeneity (HOM), contrast (CON), dissimilarity (DIS), entropy (ENT), and secondmoment (SEM).

To assess the contribution of texture information extracted from multispectral imagery, texture features were first computed separately for each spectral band (B, G, R, RE, and NIR). In addition, composite texture indices were constructed to integrate complementary texture characteristics across bands. Specifically, three types of texture indices (TIs) were derived: the normalized difference texture index (NDTI), the difference texture index (DTI), and the ratio texture index (RTI). Their formulations are provided in Equations (4)–(6), respectively.

(4) $$NDTI\left(T1,T2\right)=\left(T1-T2\right)/\left(T1+T2\right)$$

(5) $$DTI\left(T1,T2\right)=T1-T2$$

(6) $$RTI\left(T1,T2\right)=T1/T2$$

### 4.4. Measurement of Wheat Yield

At the maturity stage, grain yield was determined by harvesting wheat plants from three 1 m^2^ quadrats randomly selected from unsampled areas within each plot. The harvested samples were manually threshed, and grain yield was measured for each quadrat.

### 4.5. Data Analysis

#### 4.5.1. Machine Learning Models

Five widely used machine learning algorithms were employed to estimate crop yield based on the constructed spectral feature datasets: k-nearest neighbors (KNN), random forest (RF), extreme gradient boosting (XGBoost), support vector machine with radial basis function kernel (SVM-RBF), and Gaussian process regression with radial basis function kernel (gaussprRadial). These models were selected to represent diverse learning paradigms, including instance-based learning, ensemble tree methods, kernel-based learning, and probabilistic regression.

The KNN model estimates target values by averaging observations from the most similar samples in the feature space and serves as a simple, non-parametric baseline. In this study, the number of neighbors (k) was set to 5, and Euclidean distance was used as the similarity metric. The RF model is an ensemble learning method that constructs multiple decision trees using bootstrap sampling and random feature selection. The number of trees (ntree) was set to 500, and the number of variables randomly sampled at each split (mtry) was set to the square root of the total number of input features. XGBoost is a gradient boosting framework that sequentially fits decision trees to minimize prediction errors. The main parameters were set as follows: number of boosting rounds (nrounds) = 100, maximum tree depth (max_depth) = 6, learning rate (eta) = 0.1, subsample ratio = 0.8, and column sampling ratio (colsample_bytree) = 0.8. The SVM-RBF model maps input features into a high-dimensional space using a radial basis function kernel and performs regression by maximizing the margin. The penalty parameter (C) was set to 1, and the kernel parameter (gamma) was defined as the inverse of the number of input features. The gaussprRadial model is a probabilistic kernel-based regression approach. The radial basis kernel width parameter (sigma) was automatically estimated from the data using a median heuristic, and the model was trained under a Gaussian noise assumption. All machine learning models were implemented in the R programming environment (version 4.5.0), using the caret, randomForest, e1071, xgboost, and kernlab packages for model development and evaluation. The parameter settings were selected based on commonly adopted default values to ensure fair comparison and reproducibility across methods.

#### 4.5.2. Feature Selection Strategies and Temporal Modeling Configurations

To systematically evaluate the effects of feature composition and temporal information on yield estimation, a hierarchical modeling strategy was adopted, including single-index analysis, single-date multi-feature regression, and multi-date feature combination modeling.

(1) Single-date univariate regression analysis. As an initial diagnostic step, univariate linear regression analyses were conducted to assess the individual relationships between spectral features and yield. For each observation date, yield was regressed separately against each vegetation index (VI), color index (CI), and texture index (TI). The R^2^ was used to quantify the explanatory power of individual indices at different growth stages.

(2) Single-date multi-feature regression with different feature strategies. To evaluate the combined predictive capability of different types of spectral information at a single growth stage, multi-feature regression models were developed independently for each observation date using four feature strategies: VI-only. Vegetation indices derived from multispectral imagery; CI-only. Color indices derived from RGB imagery; TI-only. Texture indices derived from GLCM-based texture analysis; All-combined: an integrated feature set combining vegetation, color, and texture indices. For each feature category (VI, CI, and TI), features were first ranked according to their Pearson correlation coefficients with yield at the corresponding observation date. The top 10 features with the highest absolute correlations were then selected and used as predictors in the regression models. For the All-combined strategy, the top 10 features from each category (VI, CI, and TI) were jointly included, resulting in a feature set that integrates complementary spectral, color, and texture information. This correlation-based feature selection strategy was applied independently at each observation date, ensuring that the selected predictors reflected stage-specific relationships between spectral features and yield. All machine learning models were trained using these feature subsets to enable a fair comparison of different spectral information sources under a consistent modeling framework.

(3) Multi-date feature combination modeling. To further investigate the contribution of multi-temporal information to yield estimation, a multi-date modeling strategy was implemented. Six observation dates covering key growth stages were available in the dataset. From these, all possible combinations of three dates were generated, resulting in a total of 20 temporal combinations (C(6, 3) = 20, Table 5). For each temporal combination, features from the selected dates were concatenated to form multi-temporal feature vectors. The same four feature strategies (VI-only, CI-only, TI-only, and All-combined) and correlation-based feature selection approach described in Section 4.5.2-(2) were applied. Specifically, for each date within a given temporal combination, the top 10 features per category were selected based on their correlations with yield, and then aggregated across dates to construct the multi-temporal feature set. All machine learning models were subsequently trained and evaluated for each temporal combination using identical validation procedures. This design enabled a systematic assessment of how temporal feature aggregation and different spectral information sources influence yield prediction performance, while maintaining consistency with the single-date modeling framework.

#### 4.5.3. Model Construction and Performance Evaluation

All extracted features were organized into a unified plot-level dataset for subsequent modeling and analysis. Specifically, three categories of spectral features were considered: vegetation indices (VIs) derived from multispectral imagery, color indices (CIs) derived from RGB imagery, and texture indices (TIs) derived from gray-level co-occurrence matrix (GLCM)-based texture analysis. For each observation date, plot-level mean values of all indices were calculated. These features were then merged with the corresponding ground-measured yield data using plot identifiers and acquisition dates to construct the final modeling dataset. According to the experimental design, a total of 28 plot-level yield samples were collected. Descriptive statistical analysis showed that yield ranged from 3566.57 to 10,032.33 kg ha^−1^, with a mean value of 6828.92 kg ha^−1^ and a coefficient of variation of 26.23%, indicating substantial variability among plots. In addition, multi-temporal UAV imagery was used to extract a total of 28 vegetation indices, 240 texture indices, and 11 color indices for yield prediction modeling. Prior to model development, correlation analysis was conducted between each feature and yield. The top ten features from each category (VIs, TIs, and CIs), ranked by their correlation coefficients with yield, were selected for subsequent modeling. Depending on the modeling strategy, different feature subsets were constructed, including: (i) VI-based features (n = 10), (ii) CI-based features (n = 10), (iii) TI-based features (n = 10), and (iv) a combined feature set integrating all three types of indices (n = 30). This design enabled a systematic evaluation of the relative contributions of different spectral information sources to wheat yield estimation performance.

**Figure 5:**
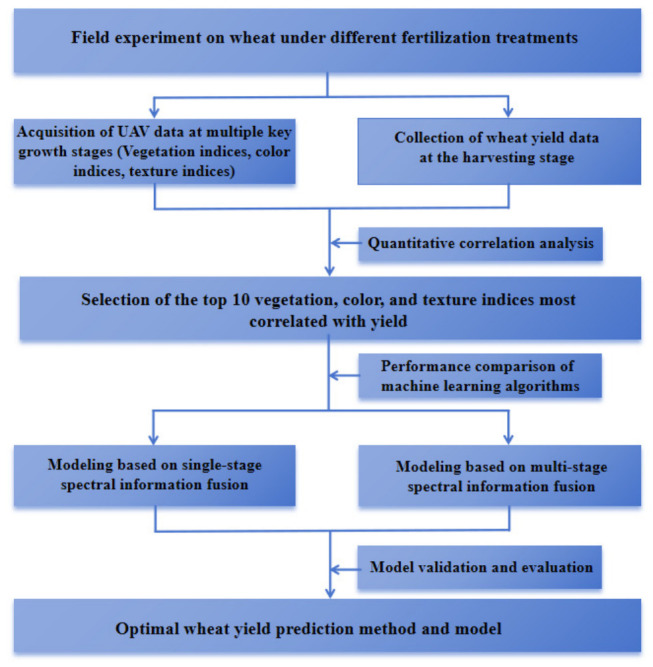
The overall technical workflow of this study.

During model training, the dataset was randomly divided into calibration (60%) and validation (40%) subsets in each iteration. This random partitioning was repeated five times to reduce potential sampling bias. To ensure reproducibility, random seeds were controlled during the splitting process. Moreover, no overlap existed between calibration and validation subsets within each iteration. Prediction accuracy was quantified using the coefficient of determination (R^2^), root mean square error (RMSE), and normalized RMSE (nRMSE). R^2^ was used to evaluate the proportion of variance in observed yield explained by the model, RMSE measured the absolute prediction error, and nRMSE was calculated by normalizing RMSE with respect to the mean observed yield to facilitate comparison across models and feature strategies. The overall technical workflow of this study is illustrated in Figure 5.

## 5. Conclusions

The results of this study demonstrate that vegetation indices derived from red-edge bands exhibit high sensitivity to wheat yield and generally outperform models based on texture and color features. Multi-feature fusion strategies further improved model performance at key phenological stages, such as the booting and flowering stages. Moreover, multi-temporal remote sensing data fusion significantly outperformed single-date modeling approaches. The yield estimation model integrating remote sensing data acquired during the late-jointing, booting and flowering stage achieved the highest prediction accuracy (R^2^ = 0.72), confirming the effectiveness of multi-temporal information in enhancing yield-related signals. Among the machine learning algorithms evaluated, XGBoost and KNN consistently exhibited superior prediction accuracy and stability across most growth stages. Overall, the integration of UAV-based multi-source and multi-temporal remote sensing data with machine learning techniques provides an effective technical pathway for high-accuracy, non-destructive wheat yield estimation.

## Figures and Tables

**Table 1:** Best-performing univariate linear regression models between yield and selected spectral, color, and texture features at each observation date.

Stage	Class	Optimal Feature	Intercept	Slope	R^2^	*p*
Pre-jointing	Spectral	EVI	4037	14,238	0.18	0.02
	Color	ExR	14,355	−126	0.11	0.09
	Texture	RTI_variance_red_edge_nir	14,501	−7740	0.08	0.57
Late-jointing	Spectral	REDVI	7099	39,012	0.15	0.04
	Color	GLI	4839	36,678	0.15	0.03
	Texture	DTI_mean_blue_green	9193	67,660	0.17	0.05
Booting	Spectral	NDRE	1971	12,089	0.41	0.01
	Color	ExGR	7283.	53.5	0.22	0.01
	Texture	DTI_mean_blue_green	12,908	129,163	0.30	0.01
Flowering	Spectral	NDRE	659	13,192	0.47	0.01
	Color	GLA	−14,245	48,294	0.29	0.01
	Texture	RTI_variance_red_edge_nir	12,888	−9600	0.21	0.01
Pre-filling	Spectral	NDRE	5860	24,793	0.13	0.06
	Color	B_mean	12,035	−101	0.25	0.01
	Texture	DTI_mean_blue_green	12,048	140,457	0.34	0.00
Late-filling	Spectral	NDRE	5123	43,711	0.17	0.02
	Color	GLA	40,742	−92,794	0.28	0.01
	Texture	DTI_variance_blue_green	9649	171	0.14	0.05

**Table 2:** Best-performing single-date regression results under different feature strategies.

Date	Strategy	Model	R^2^	RMSE	nRMSE	Num_Features	Computation Time (s)	Memory Usage (MB)
Pre-jointing	Spectral	XGBoost	0.18	2032.91	0.29	10	0.24	58.40
	Color	XGBoost	0.19	2372.38	0.34	10	0.23	57.90
	Texture	SVM-RBF	0.16	2181.64	0.31	10	0.31	61.70
	All	XGBoost	0.15	2326.08	0.34	30	0.32	73.60
Late-jointing	Spectral	SVM-RBF	0.15	1836.75	0.26	10	0.31	60.80
	Color	gaussprRadial	0.23	682.24	0.24	10	0.33	70.50
	Texture	XGBoost	0.19	2031.17	0.29	10	0.25	58.70
	All	KNN	0.19	1833.39	0.26	30	0.30	69.20
Booting	Spectral	XGBoost	0.49	1263.13	0.17	10	0.21	56.80
	Color	KNN	0.25	1724.92	0.25	10	0.14	49.60
	Texture	XGBoost	0.25	1757.29	0.25	10	0.22	57.40
	All	XGBoost	0.67	993.45	0.14	30	0.28	74.80
Flowering	Spectral	XGBoost	0.31	1678.24	0.24	10	0.20	56.90
	Color	KNN	0.25	1715.48	0.25	10	0.15	49.90
	Texture	XGBoost	0.33	1701.63	0.24	10	0.22	57.60
	All	KNN	0.53	1159.94	0.17	30	0.23	69.80
Pre-filling	Spectral	KNN	0.19	1751.63	0.25	10	0.13	48.80
	Color	RF	0.27	1676.68	0.24	10	0.17	54.300
	Texture	XGBoost	0.23	1887.80	0.27	10	0.23	57.80
	All	RF	0.27	1661.50	0.24	30	0.24	69.40
Late-filling	Spectral	SVM-RBF	0.11	1831.10	0.26	10	0.30	61.20
	Color	XGBoost	0.30	1572.63	0.23	10	0.21	56.50
	Texture	XGBoost	0.18	1917.72	0.28	10	0.22	57.30
	All	XGBoost	0.21	1736.01	0.25	30	0.31	74.10

**Table 3:** Top-performing combinations of temporal windows, feature strategies, and machine-learning models for multi-date yield estimation. Notes: PJS, LJS, BS, FS, PFS and LFS represent the Pre-jointing, Late-jointing, Booting, Flowering, Pre-filling, and Late-filling stage, respectively.

Dates	Strategy	Model	R^2^	RMSE	nRMSE	Num_Features	Computation Time (s)	Memory Usage (Mb)
LJS, BS, FS	Spectral	XGBoost	0.72	915.00	0.13	30	0.56	88.60
LJS, BS, PFS	Spectral	XGBoost	0.70	964.00	0.15	30	0.55	87.90
BS, FS, PFS	Spectral	XGBoost	0.66	1047.00	0.15	30	0.52	86.70
LJS, BS, LFS	Color	XGBoost	0.33	1530.00	0.22	30	0.49	85.80
LJS, FS, LFS	Color	XGBoost	0.28	1650.00	0.24	30	0.47	84.90
LJS, BS, FS	Color	KNN	0.28	1716.00	0.25	30	0.34	71.40
PJS, LJS, PFS	Texture	XGBoost	0.43	1631.00	0.24	30	0.50	86.10
PJS, BS, PFS	Texture	XGBoost	0.41	1612.00	0.24	30	0.48	85.60
LJS, BS, FS	Texture	SVM-RBF	0.39	1508.00	0.22	30	0.61	90.80
LJS, BS, FS	All	XGBoost	0.54	1204.00	0.17	90	0.96	132.40
BS, FS, LFS	All	XGBoost	0.48	1343.00	0.20	90	0.89	128.70
PJS, BS, LFS	All	XGBoost	0.46	1330.00	0.19	90	0.92	130.60

**Table 4:** Spectral indices and their mathematical formulations.

Spectral index	Formula	Reference
Vegetation index		
Ratio vegetation index (BRVI)	Nir/B	[36]
Normalized difference vegetation index (BNDVI)	(Nir − B)/(Nir + B)	[37]
Difference vegetation index (BDVI)	Nir − B	[38]
Re-normalized different vegetation index (BRDVI)	(Nir − B)/SQRT(Nir + B)	[39]
Soil-adjusted vegetation index (BSAVI)	1.5 × (Nir − B)/(Nir + B +0.5)	[40]
Optimized soil-adjusted vegetation index (BOSAVI)	(Nir − B)/(Nir + B +0.16)	[41]
Wide dynamic range vegetation index (BWDRVI)	(0.12 × Nir − B)/(0.12 × Nir + B)	[42]
Green ratio vegetation index (GRVI)	Nir/G	[37]
Normalized difference vegetation index (GNDVI)	(Nir − G)/(Nir + G)	[43]
Difference vegetation index (GDVI)	Nir − G	[44]
Re-normalized different vegetation index (GRDVI)	(Nir − G)/SQRT(Nir + G)	[44]
Soil-adjusted vegetation index (GSAVI)	1.5 × (Nir − G)/(Nir + G + 0.5)	[44]
Optimized soil-adjusted vegetation index (GOSAVI)	(Nir − G)/(Nir + G + 0.16)	[44]
Wide dynamic range vegetation index (GWDRVI)	(0.12 × Nir − G)/(0.12 × Nir + G)	[44]
Ratio vegetation index (RVI)	Nir/R	[36]
Normalized difference vegetation index (NDVI)	(Nir − R)/(Nir + R)	[45]
Difference vegetation index (DVI)	Nir − R	[38]
Re-normalized different vegetation index (RDVI)	(Nir − R)/SQRT(Nir + R)	[39]
Soil-adjusted vegetation index (SAVI)	1.5 × (Nir − R)/(Nir + R + 0.5)	[40]
Optimized soil-adjusted vegetation index (OSAVI)	(Nir − R)/(Nir + R + 0.16)	[41]
Wide dynamic range vegetation index (WDRVI)	(0.12 × Nir − R)/(0.12 × Nir + R)	[42]
Ratio vegetation index (RERVI)	Nir/RE	[46]
Normalized difference vegetation index (NDRE)	(Nir − RE)/(Nir + RE)	[47]
Difference vegetation index (REDVI)	Nir − RE	[48]
Re-normalized different vegetation index (RERDVI)	(Nir − RE)/SQRT(Nir + RE)	[49]
Soil-adjusted vegetation index (RESAVI)	1.5 × (Nir − RE)/(Nir + RE + 0.5)	[48]
Optimized soil-adjusted vegetation index (REOSAVI)	(Nir − RE)/(Nir + RE + 0.16)	[48]
Wide dynamic range vegetation index (REWDRVI)	(0.12 × Nir − RE)/(0.12 × Nir + RE)	[42]
Color index		
Normalized green minus red difference index (NGRDI)	(g − r)/(g + r)	[50]
Normalized green minus blue difference index (NGBDI)	(g − b)/(g + b)	[51]
Excess green index (ExG)	2g − r − b	[50]
Excess red index (ExR)	1.4r − g	[52]
Excess green minus excess red index (ExGR)	3g − 2.4r − b	[53]
Visible atmospherically resistant index (VARI)	(g − r)/(g + r − b)	[52]
Green leaf index (GLI)	(2g − b − r)/(2g + b + r)	[54]
Modified green-red vegetation index (MGRVI)	(g2 − r2)/(g2 + r2)	[55]
Green-red vegetation index (GRVI)	(g − r)/(g + r)	[55]
Green leaf algorithm (GLA)	(2g − r + b)/(2g + r + b)	[54]
Red green blue vegetation index (RGBVI)	(g × r − r × b)/(g × g + r × b)	[56]

**Table 5:** All possible date combinations derived from six observation dates.

Number	Dates Combination
1	Pre-jointing, Late-jointing, Booting
2	Pre-jointing, Late-jointing, Flowering
3	Pre-jointing, Late-jointing, Pre-filling
4	Pre-jointing, Late-jointing, Late-filling
5	Pre-jointing, Booting, Flowering
6	Pre-jointing, Booting, Pre-filling
7	Pre-jointing, Booting, Late-filling
8	Pre-jointing, Flowering, Pre-filling
9	Pre-jointing, Flowering, Late-filling
10	Pre-jointing, Pre-filling, Late-filling
11	Late-jointing, Booting, Flowering
12	Late-jointing, Booting, Pre-filling
13	Late-jointing, Booting, Late-filling
14	Late-jointing, Flowering, Pre-filling
15	Late-jointing, Flowering, Late-filling
16	Late-jointing, Pre-filling, Late-filling
17	Booting, Flowering, Pre-filling
18	Booting, Flowering, Late-filling
19	Booting, Pre-filling, Late-filling
20	Flowering, Pre-filling, Late-filling

## Data Availability

The data used in this study are available from the corresponding authors upon request.

## References

[1] Li Q., Jin S., Zang J., Wang X., Sun Z., Li Z., Xu S., Ma Q., Su Y., Guo Q. (2022). Deciphering the contributions of spectral and structural data to wheat yield estimation from proximal sensing. Crop J..

[2] Korohou T., Okinda C., Li H., Cao Y., Nyalala I., Huo L., Potcho M., Li X., Ding Q. (2020). Wheat grain yield estimation based on image morphological properties and wheat biomass. J. Sens..

[3] Mathan J., Bhattacharya J., Ranjan A. (2016). Enhancing crop yield by optimizing plant developmental features. Development.

[4] Feng L., Wang Y., Zhang Z., Du Q. (2021). Geographically and temporally weighted neural network for winter wheat yield prediction. Remote Sens. Environ..

[5] Cai Y., Guan K., Lobell D., Potgieter A.B., Wang S., Peng J., Xu T., Asseng S., Zhang Y., You L. (2019). Integrating satellite and climate data to predict wheat yield in Australia using machine learning approaches. Agric. For. Meteorol..

[6] Guan K., Wu J., Kimball J.S., Anderson M.C., Frolking S., Li B., Hain C.R., Lobell D.B. (2017). The shared and unique values of optical, fluorescence, thermal and microwave satellite data for estimating large-scale crop yields. Remote Sens. Environ..

[7] Li B., Liu R., Liu S., Liu Q., Liu F., Zhou G. (2012). Monitoring vegetation coverage variation of winter wheat by low-altitude UAV remote sensing system. Trans. Chin. Soc. Agric. Eng..

[8] Zhu W., Li S., Zhang X., Li Y., Sun Z. (2018). Estimation of winter wheat yield using optimal vegetation indices from unmanned aerial vehicle remote sensing. Trans. Chin. Soc. Agric. Eng..

[9] Zhou X., Zheng H., Xu X., He J., Ge X., Yao X., Cheng T., Zhu Y., Cao W., Tian Y. (2017). Predicting grain yield in rice using multi-temporal vegetation indices from UAV-based multispectral and digital imagery. ISPRS J. Photogramm. Remote Sens..

[10] Gong Y., Duan B., Fang S., Zhu R., Wu X., Ma Y., Peng Y. (2018). Remote estimation of rapeseed yield with unmanned aerial vehicle (UAV) imaging and spectral mixture analysis. Plant Methods.

[11] Aula L., Omara P., Nambi E., Oyebiyi F.B., Dhillon J., Eickhoff E., Carpenter J., Raun W.R. (2021). Active optical sensor measurements and weather variables for predicting winter wheat yield. Agron. J..

[12] Du M., Noguchi N. (2017). Monitoring of wheat growth status and mapping of wheat yield’s within-field spatial variations using color images acquired from UAV-camera system. Remote Sens..

[13] Wood E.M., Pidgeon A.M., Radeloff V.C., Keuler N.S. (2012). Image texture as a remotely sensed measure of vegetation structure. Remote Sens. Environ..

[14] Guo C., Tang Y., Lu J., Zhu Y., Cao W., Cheng T., Zhang L., Tian Y. (2019). Predicting wheat productivity: Integrating time series of vegetation indices into crop modeling via sequential assimilation. Agric. For. Meteorol..

[15] Mateo-Sanchis A., Piles M., Muñoz-Marí J., Adsuara J.E., Pérez-Suay A., Camps-Valls G. (2019). Synergistic integration of optical and microwave satellite data for crop yield estimation. Remote Sens. Environ..

[16] Ashapure A., Jung J., Chang A., Oh S., Yeom J., Maeda M., Maeda A., Dube N., Landivar J., Hague S. (2020). Developing a machine learning based cotton yield estimation framework using multi-temporal UAS data. ISPRS J. Photogramm. Remote Sens..

[17] Wang L., Zhou X., Zhu X., Dong Z., Guo W. (2016). Estimation of biomass in wheat using random forest regression algorithm and remote sensing data. Crop J..

[18] Maimaitijiang M., Sagan V., Sidike P., Hartling S., Esposito F., Fritschi F.B. (2020). Soybean yield prediction from UAV using multimodal data fusion and deep learning. Remote Sens. Environ..

[19] Fu Z., Jiang J., Gao Y., Krienke B., Wang M., Zhong K., Cao Q., Tian Y., Zhu Y., Cao W. (2020). Wheat growth monitoring and yield estimation based on multi-rotor unmanned aerial vehicle. Remote Sens..

[20] White J.W., Andrade-Sanchez P., Gore M.A., Bronson K.F., Coffelt T.A., Conley M.M., Feldmann K.A., French A.N., Heun J.T., Hunsaker D.J. (2012). Field-based phenomics for plant genetics research. Field Crops Res..

[21] Gong T., Zhu X., Chen Y., Xiong S. (2025). Temporal-aware spatial interaction transformer for crop yield prediction based on multi-sensor satellite image time series. IEEE Geosci. Remote Sens. Lett..

[22] Jhajharia K. (2025). Wheat yield prediction of Rajasthan using climatic and satellite data and machine learning techniques. J. Agrometeorol..

[23] Fathololoumi S., Vasava H., Firozjaei M.K., Daggupati P., Sulik J., Biswas A. (2025). Reducing corn yield prediction uncertainty through multi-scale integration of ground, drone, and satellite data. Precis. Agric..

[24] Tao H., Xu S., Tian Y., Li Z., Ge Y., Zhang J., Wang Y., Zhou G., Deng X., Zhang Z. (2022). Proximal and remote sensing in plant phenomics: 20 years of progress, challenges, and perspectives. Plant Commun..

[25] Wang X., Guo P., Li X., Gangopadhyay A., Busart C.E., Freeman J., Wang J. (2023). Reproducible and portable big data analytics in the cloud. IEEE Trans. Cloud Comput..

[26] Kanke Y., Raun W., Solie J., Stone M., Taylor R. (2012). Red edge as a potential index for detecting differences in plant nitrogen status in winter wheat. J. Plant Nutr..

[27] Jiang J., Zhang Z., Cao Q., Liang Y., Krienke B., Tian Y., Zhu Y., Cao W., Liu X. (2020). Use of an active canopy sensor mounted on an unmanned aerial vehicle to monitor the growth and nitrogen status of winter wheat. Remote Sens..

[28] Zhang J., Qiu X., Wu Y., Zhu Y., Cao Q., Liu X., Cao W. (2021). Combining texture, color, and vegetation indices from fixed-wing UAS imagery to estimate wheat growth parameters using multivariate regression methods. Comput. Electron. Agric..

[29] Zheng H., Ma J., Zhou M., Li D., Yao X., Cao W., Zhu Y., Cheng T. (2020). Enhancing the nitrogen signals of rice canopies across critical growth stages through the integration of textural and spectral information from unmanned aerial vehicle (UAV) multispectral imagery. Remote Sens..

[30] Yu X., Yin D., Xu H., Pinto Espinosa F., Schmidhalter U., Nie C., Bai Y., Sankaran S., Ming B., Cui N. (2024). Maize tassel number and tasseling stage monitoring based on near-ground and UAV RGB images by improved YOLOv8. Precis. Agric..

[31] Liu Y., Feng H., Yue J., Jin X., Fan Y., Chen R., Bian M., Ma Y., Song X., Yang G. (2023). Improved potato AGB estimates based on UAV RGB and hyperspectral images. Comput. Electron. Agric..

[32] Sahbeni G., Székely B., Musyimi P.K., Timár G., Sahajpal R. (2023). Crop yield estimation using Sentinel-3 SLSTR, soil data, and topographic features combined with machine learning modeling: A case study of Nepal. AgriEngineering.

[33] Diacono M., Rubino P., Montemurro F. (2013). Precision nitrogen management of wheat: A review. Agron. Sustain. Dev..

[34] Zhang Y., Han X., Yang J. (2025). Evaluation of spectral features and regression methods for leaf area index estimation. Eur. J. Remote Sens..

[35] Dhillon J., Aula L., Eickhoff E., Raun W. (2020). Predicting in-season maize (*Zea mays* L.) yield potential using crop sensors and climatological data. Sci. Rep..

[36] Pearson R.L., Miller L.D. Remote mapping of standing crop biomass for estimation of the productivity of the shortgrass prairie, Pawnee National Grasslands, Colorado. Proceedings of the Eighth International Symposium on Remote Sensing of Environment.

[37] Buschmann C., Nagel E. (1993). In vivo spectroscopy and internal optics of leaves as basis for remote sensing of vegetation. Int. J. Remote Sens..

[38] Richardson A.J., Weigand C.L. (1977). Distinguishing vegetation from soil background information. Photogramm. Eng. Remote Sens..

[39] Guyot G., Baret F. (1988). Utilisation de la haute resolution spectrale pour suivre l’etat des couverts vegetaux. Spectr. Signat. Objects Remote Sens..

[40] Huete A.R. (1988). A soil-adjusted vegetation index (SAVI). Remote Sens. Environ..

[41] Rondeaux G., Steven M., Baret F. (1996). Optimization of soil-adjusted vegetation indices. Remote Sens. Environ..

[42] Gitelson A.A. (2004). Wide dynamic range vegetation index for remote quantification of biophysical characteristics of vegetation. J. Plant Physiol..

[43] Gitelson A.A., Kaufman Y.J., Merzlyak M.N. (1996). Use of a green channel in remote sensing of global vegetation from EOS-MODIS. Remote Sens. Environ..

[44] Huang S., Miao Y., Zhao G., Yuan F., Ma X., Tan C., Yu W., Gnyp M.L., Lenz-Wiedemann V.I., Rascher U. (2015). Satellite remote sensing-based in-season diagnosis of rice nitrogen status in Northeast China. Remote Sens..

[45] Tucker C.J. (1979). Red and photographic infrared linear combinations for monitoring vegetation. Remote Sens. Environ..

[46] Gitelson A.A., Merzlyak M.N., Lichtenthaler H.K. (1996). Detection of red edge position and chlorophyll content by reflectance measurements near 700 nm. J. Plant Physiol..

[47] Barnes M., Clarke T.R., Richards S.E., Colaizzi P., Haberland J., Kostrzewski M., Waller P., Choi C., Riley E., Thompson T. Coincident detection of crop water stress, nitrogen status, and canopy density using ground-based multispectral data. Proceedings of the Fifth International Conference on Precision Agriculture.

[48] Cao Q., Miao Y., Wang H., Huang S., Cheng S., Khosla R., Jiang R. (2013). Non-destructive estimation of rice plant nitrogen status with Crop Circle multispectral active canopy sensor. Field Crops Res..

[49] Roujean J.-L., Breon F.-M. (1995). Estimating PAR absorbed by vegetation from bidirectional reflectance measurements. Remote Sens. Environ..

[50] Woebbecke D.M., Meyer G.E., Von Bargen K., Mortensen D.A. (1995). Color indices for weed identification under various soil, residue, and lighting conditions. Trans. ASAE.

[51] Hunt E.R., Cavigelli M., Daughtry C.S.T., McMurtrey J.E., Walthall C.L. (2005). Evaluation of digital photography from model aircraft for remote sensing of crop biomass and nitrogen status. Precis. Agric..

[52] Meyer G.E., Hindman T.W., Laksmi (1999). Machine vision detection parameters for plant species identification. Precis. Agric..

[53] Meyer G.E., Neto J.C. (2008). Verification of color vegetation indices for automated crop imaging applications. Comput. Electron. Agric..

[54] Louhaichi M., Borman M.M., Johnson D.E. (2001). Spatially located platform and aerial photography for documentation of grazing impacts on wheat. Geocarto Int..

[55] Bendig J., Yu K., Aasen H., Bolten A., Bennertz S., Broscheit J., Gnyp M.L., Bareth G. (2015). Combining UAV-based plant height from crop surface models, visible, and near infrared vegetation indices for biomass monitoring in barley. Int. J. Appl. Earth Obs. Geoinf..

[56] Li C., Niu Q., Yang G., Feng H., Liu J., Wang Y. (2017). Estimation of leaf area index of soybean breeding materials based on UAV digital images. Trans. Chin. Soc. Agric. Mach..

